# Effect of Lactation on myocardial vulnerability to ischemic insult in rats

**DOI:** 10.5935/abc.20170042

**Published:** 2017-05

**Authors:** Sahar Askari, Alireza Imani, Hamidreza Sadeghipour, Mahdieh Faghihi, Zohreh Edalatyzadeh, Samira Choopani, Nasser Karimi, Sulail Fatima

**Affiliations:** 1 Tehran University of Medical Sciences, Tehran, Iran; 2 Rassoul Akram Hospital - Iran University of Medical Sciences, Tehran, Iran; 3 Tehran University of Medical Sciences - International Campus, Tehran, Iran

**Keywords:** Lactation, Myocardial Infarction, Myocardial Ischemia, Parturitium

## Abstract

**Background:**

Cardiovascular diseases are the leading cause of mortality and long-term disability worldwide. Various studies have suggested a protective effect of lactation in reducing the risk of cardiovascular diseases.

**Objective:**

This study was designed to assess the effects of pregnancy and lactation on the vulnerability of the myocardium to an ischemic insult.

**Methods:**

Eighteen female rats were randomly divided into three groups: ischemia-reperfusion (IR), in which the hearts of virgin rats underwent IR (n = 6); lactating, in which the rats nursed their pups for 3 weeks and the maternal hearts were then submitted to IR (n = 6); and non-lactating, in which the pups were separated after birth and the maternal hearts were submitted to IR (n = 6). Outcome measures included heart rate (HR), left ventricular developed pressure (LVDP), rate pressure product (RPP), ratio of the infarct size to the area at risk (IS/AAR %), and ventricular arrhythmias - premature ventricular contraction (PVC) and ventricular tachycardia (VT).

**Results:**

The IS/AAR was markedly decreased in the lactating group when compared with the non-lactating group (13.2 ± 2.5 *versus* 39.7 ± 3.5, p < 0.001) and the IR group (13.2 ± 2.5 *versus* 34.0 ± 4.7, p < 0.05). The evaluation of IR-induced ventricular arrhythmias indicated that the number of compound PVCs during ischemia, and the number and duration of VTs during ischemia and in the first 5 minutes of reperfusion in the non-lactating group were significantly (p < 0.05) higher than those in the lactating and IR groups.

**Conclusion:**

Lactation induced early-onset cardioprotective effects, while rats that were not allowed to nurse their pups were more susceptible to myocardial IR injury.

## Introduction

Coronary artery diseases are the leading cause of mortality worldwide, with about 38% of the deaths attributed to these diseases.^1^ In addition to hypertension and diabetes, multiple lifestyle factors, including a high-cholesterol diet, smoking, alcohol consumption, and stress, can increase the risk of myocardial infarction.^2^ Accumulating epidemiological evidence suggests that a woman's decision to breast-feed her children has a significant impact on the maternal risk of developing cardiovascular diseases.^3,4^ Although national policies to promote breastfeeding have been profoundly implemented in many developed countries, the global rate of exclusive breastfeeding is below 40%.^5^

Lactation confers significant benefits to the maternal cardiovascular health.^6^ Lactogenesis has a favorable effect on glucose and lipid metabolism. It increases insulin sensitivity and glucose effectiveness while reducing the risk of type 2 diabetes.^3^ Nursing promotes high-density lipoprotein production and reduces triglyceride and low-density lipoprotein levels.^7,8^ Consequently, lactation mobilizes the fat stores generated during pregnancy, thereby reducing the risk of cardiovascular diseases and myocardial infarction.^9^ Furthermore, lactation reduces maternal blood pressure and heart rate (HR), while improving cardiac output.^10^ Hanwell and Peaker^11^ observed that the augmented cardiac output is directly proportional to the intensity of suckling in rats.^11^ Moreover, certain hormones released during lactation, such as oxytocin, prolactin, glucocorticoids, gherlin and growth hormone, can precondition the myocardium against cardiac injury.^12,13^ Centrally released endogenous oxytocin and exogenous infusion of oxytocin have been shown to protect heart against a hypoxic insult via activation of brain receptors.^14^ Furthermore, oxytocin induces cardioprotection through a pathway involving mitochondrial ATP-dependent potassium channels.^15^ Although, the effect of lactogenic hormones on cardiovascular health has been studied to some extent, the experimental evidence suggesting the cardioprotective role of lactation against ischemia-reperfusion (IR) injury is scarce. Thus, the current was designed to test the hypothesis that lactation reduces the myocardial vulnerability to IR injury.

## Methods

### Animals

Eighteen female Sprague-Dawley rats (180-230 g) were housed in an air-conditioned colony room at 21-23°C, with a 12-hour light-dark cycle. During the experimental period, the animals had free access to food and water. The experimental protocols followed in this study conformed to the Guidelines for the Care and Use of Laboratory Animals published by National Institutes of Health (NIH Publication N°. 85-23, revised 1996) and were further approved by the institutional ethical committee at Tehran University of Medical Sciences (Tehran, Iran).

### Preparation of isolated hearts

The animals were anesthetized using intraperitoneal sodium thiopental (60 mg/kg). Heparin (500 IU/kg) was also injected to prevent blood coagulation. Once the animals were anesthetized, a transabdominal incision was made, and their hearts were exposed. Following cannulation of the aorta, the heart was excised and mounted on a Langendorff apparatus. The hearts were perfused retrogradely with Krebs-Henseleit bicarbonate buffer containing (in mmol/L): NaHCO_3_ 25; KCl 4.7; NaCl 118.5; MgSO_4_ 1.2; KH_2_PO_4_ 1.2; glucose 11; CaCl_2_ 2.5, gassed with 95% O_2_ and 5% CO_2_ (pH 7.35-7.45 at 37 C°). A saline-filled latex balloon was introduced into the left ventricle and inflated to yield a preload of 8-10 mmHg. The balloon was connected to a pressure transducer (Harvard, March-Hugsteten, Germany) which allowed a real time measurement of the pressures from the ventricle. Electrocardiographic recording was performed by fixation of thin electrodes on the ventricular apex and right atrium. A surgical needle (6-0 silk suture) was passed under the origin of the left anterior descending coronary artery, and the ends of the suture were passed through two plastic pipette tips to form a snare. Regional ischemia was induced by tightening the snare (30 min), and reperfusion was performed by releasing the ends of the suture (60 min). The hearts were allowed to beat spontaneously throughout the experiments.

### Sample size estimation

The sample size was estimated using the Resource Equation Method.^16^

E = N - T

E = degrees of freedom (analysis of variance [ANOVA]; between 10 and 20)

N = total number of animals

T = number of treatment groups

"N" was obtained from a previously published study^17^ and was adjusted accordingly to attain a valid "E".

### Experimental groups

The effects of pregnancy and lactation on myocardial IR injury were studied in 18 rats randomly divided into three groups:

1. IR group (IR; n = 6): isolated hearts of virgin rats in the diestrus period underwent 30 min of regional ischemia followed by 60 min of reperfusion.
2. Lactating group (n = 6): the rats nursed their pups for 3 weeks and, after that, the maternal hearts underwent 30 min of regional ischemia followed by 60 min of reperfusion.
3. Non-lactating group (n = 6): after parturition, the rats were separated from their pups, and 3 weeks later, the maternal hearts underwent 30 min of regional ischemia followed by 60 min of reperfusion.

### Hemodynamic functions

The left ventricular developed pressure (LVDP) and the HR were continuously monitored and recorded using BioLab data acquisition system. The rate pressure product (RPP) was calculated by multiplying LVDP by the HR.

### Assessment of area at risk and infarct size

At the end of reperfusion, the left coronary artery was reoccluded and Evans blue (0.3-0.5 ml) dye was infused via aorta to differentiate the ischemic zone (area at risk; AAR) from the non-ischemic zone. The hearts were frozen at - 20°C and sliced into 2.0 mm traverse sections (using a stainless steel slicer matrix with 2.0 mm coronal section slice intervals) from apex to the base. The slices were incubated in 1% triphenyltetrazolium chloride (TTC in 0.1 M phosphate buffer, pH 7.4, 37 ºC) for 20 min followed by tissue fixation (10% phosphate-buffered formalin) for 24 h. TTC reacts with the viable tissue, producing a red formazan derivative which is distinct from the white necrotic area. Sections were scanned to determine non-ischemic area, AAR (ischemic area) and the infarct size (IS) by calculating the pixels occupied by each area using the Adobe PhotoShop software (Adobe Systems Seattle, WA). The AAR was expressed as a percentage of left ventricular volume for each heart. The IS was determined by using computer-aided planimetry and expressed as a percentage of the AAR.^18-20^

### Assessment of ventricular arrhythmias

Ischemia-induced ventricular arrhythmias were assessed during the occlusion period and were determined in accordance with the Lambeth Conventions.^21^ Ventricular tachycardia (VT) and premature ventricular contraction (PVC) including compound PVCs (such as bigeminy, couplet and salvos) were counted during ischemic period and the first 5 minutes of the reperfusion period.

### Statistical analysis

All data were statistically analyzed using GraphPad InStat, version 3.06 (GraphPad Software, Inc., San Diego, CA). The data followed Gaussian distribution (Kolmogorov-Smirnov test). All results are expressed as mean ± standard error of the mean (SEM). Outcome measures between the groups were analyzed using one-way ANOVA followed by the Bonferroni *post hoc* test. For intragroup comparisons, repeated measures ANOVA followed by the Bonferroni *post hoc* test (for selected columns) was performed. Statistical significance was defined as p < 0.05.

## Results

### Hemodynamic Parameters

Table 1 demonstrates the changes in HR, LVDP, and RPP in the IR, lactating and non-lactating groups during different periods of the experiment.

**Table 1:** Hemodynamic Parameters

Groups	Baseline			Ischemia			Reperfusion		
HR (Beats/min)	LVDP (mmHg)	RPP (mmHg x bpm)	HR (Beats/min)	LVDP (%Baseline)	RPP (%Baseline)	HR (Beats/min)	LVDP (%Baseline)	RPP (%Baseline)
IR	146.0 ± 4.9	58.4 ± 11.9	8519.6 ± 1805.2	134.6 ± 3.9	62.5 ± 8.3^*^	57.5 ± 7.3^#^	131.2 ± 5.2^$^	64.3 ± 8.6^*^^$^	57.4 ± 6.8^#^
L	138.0 ± 3.6	115.2 ± 34.2	15673.2 ± 4219.6	120.7 ± 9.1	28.3 ± 3.7^*^	25.2 ± 4.6^*^	71.7 ± 18.4	20.8 ± 7.8^#^	12.6 ± 7.2^#^
NL	148.6 ± 8.3	66.9 ± 11.9	9797.3 ± 1652.5	137.4 ± 9.7	73 ± 8.5^$^	67.2 ± 7.7.^*^^$^	145.4 ± 11.9^$^	73.1 ± 8.5^$^	74.0 ± 13^$^

* p < 0.05 versus the baseline period in the group,

# p < 0.001 versus the baseline period in the group,

$ p < 0.05 versus the lactating group.

Data are presented as mean ± standard error of the mean (SEM). For LVDP, RPP and HR, the means were compared between the groups using one-way ANOVA and within the groups using repeated measure ANOVA. The post hoc test used was Bonferroni. IR: ischemia-reperfusion; L: lactating; NL: non-lactating; LVDP: left ventricular developed pressure (mmHg); HR: heart rate (beats/min), RPP: rate pressure product (mmHg x bpm); bpm: beats per minute

In IR group, LVDP and RPP were reduced (p < 0.05 and p < 0.001, respectively) at the end of ischemia and reperfusion period as compared with the baseline. In lactating group, LVDP and RPP reduced significantly at the end of the ischemia (p < 0.05) and reperfusion (p < 0.001) periods when compared with baseline. Furthermore, in the lactating group, the HR was markedly reduced in the reperfusion period when compared with baseline. Non-lactating animals demonstrated lower RPP during ischemia when compared with baseline (p < 0.05).

Intergroup analysis showed that LVDP at the end of ischemia, and RPP in the ischemia and reperfusion periods were significantly higher in the non-lactating when compared to the lactating group (p < 0.05). In addition, HR and LVDP in reperfusion were markedly increased in the IR and non-lactating groups, when compared with the lactating group (p < 0.05).

### Area at risk and infarct size

**Figure 1:**
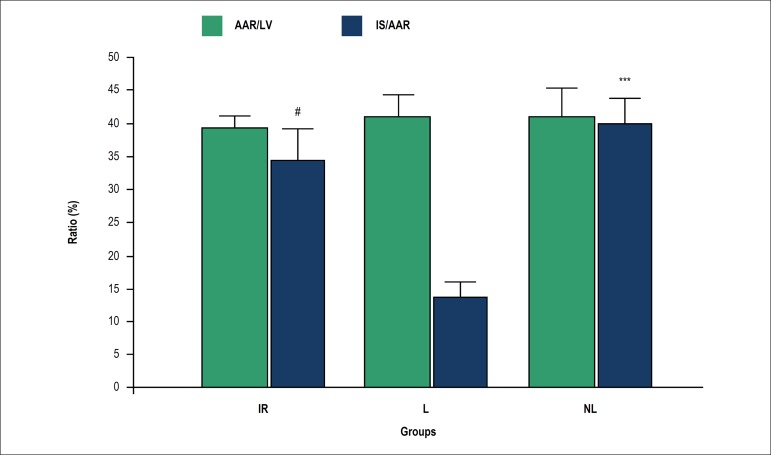
Myocardial area at risk in relation to the left ventricle (AAR/V %) and infarct size (IS/AAR %). Data are presented as mean ± S.E.M. standard error of the mean (SEM). The mean values between the groups were compared using one-way ANOVA followed by Bonferroni's post hoc test. # p < 0.05 versus the lactating group, *** p < 0.001 versus the lactating group. IR: ischemia-reperfusion; L: lactating; NL: Non-lactating.

As shown in Figure 1, there was no significant difference in AAR between the groups. However, the IS/AAR was significantly reduced in the lactating group as compared with the non-lactating and IR groups (13.2 ± 2.5 *versus* 39.7 ± 3.5 and 34.0 ± 4.7, respectively).

### Ventricular arrhythmias

**Figure 2:**
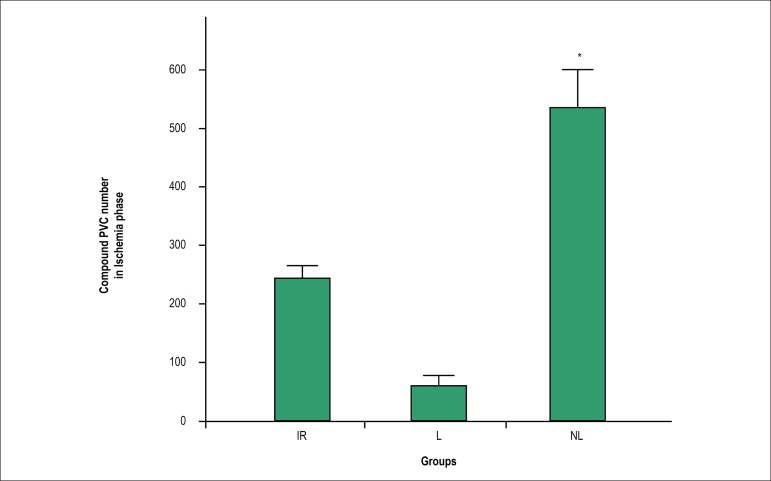
Number of compound PVCs (including bigeminy, couplet and salvos) in different groups. Data are presented as mean ± standard error of the mean (SEM). The mean values between the groups were compared using one-way ANOVA followed by Bonferroni's post hoc test. * p < 0.05 versus the lactating group. IR: ischemia-reperfusion; L: lactating; NL: Non-lactating.

**Figure 3:**
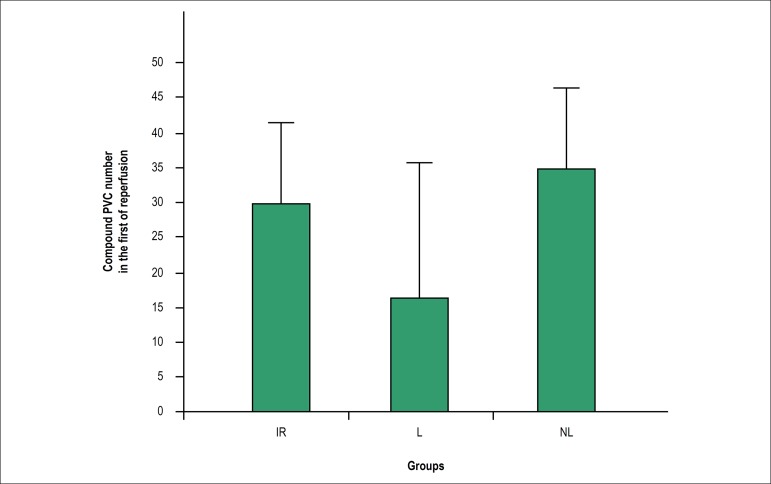
Number of compound PVCs (including bigeminy, couplet and salvos) in the first 5 min of reperfusion. Data are presented as mean ± standard error of the mean (SEM). The mean values between the groups were compared using one-way ANOVA followed by Bonferroni's post hoc test. IR: ischemia-reperfusion; L: lactating; NL: Non-lactating.

**Figure 4:**
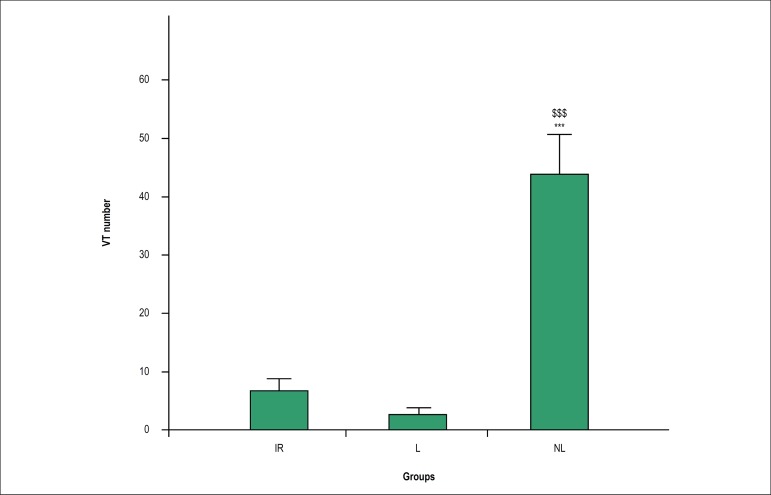
Number of ventricular tachycardia (VT) in different groups. Data are presented as mean ± standard error of the mean (SEM). The mean values between the groups were compared using one-way ANOVA followed by Bonferroni's post hoc test. *** p < 0.001 versus the lactating group; $$$ p < 0.001 versus the IR group. IR: ischemia-reperfusion; L: lactating; NL: Non-lactating.

**Figure 5:**
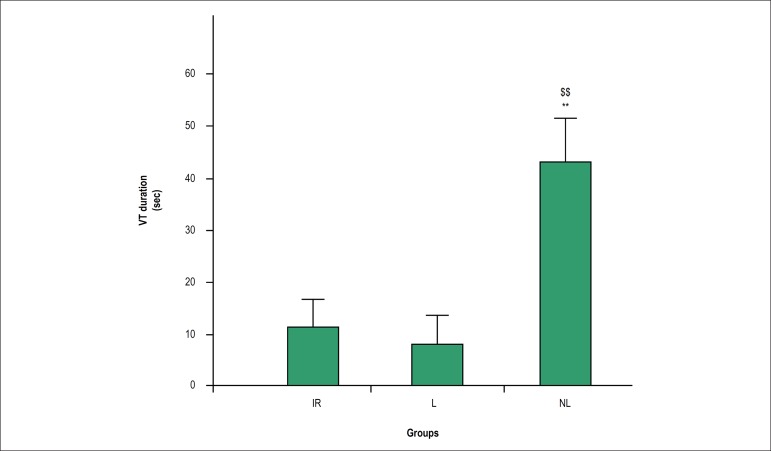
Duration of ventricular tachycardia (VT) in different groups. Data are presented as mean ± standard error of the mean (SEM). The mean values between the groups were compared using one-way ANOVA followed by Bonferroni's post hoc test. IR: ischemia-reperfusion; L: lactating; NL: non-lactating. ** p < 0.001 versus the lactating group; $$ p < 0.001 versus the IR group.

During the ischemic phase, the number of compound PVCs was statistically higher in the non-lactating group as compared with the lactating group (p < 0.05) (Figure 2). During the first 5 min of the reperfusion phase, the number of compound PVCs did not differ significantly between the groups (Figure 3). During ischemia and the first 5 min of reperfusion, the number of VTs was significantly lower in the lactating and IR groups as compared with the non-lactating group (p < 0.001) (Figure 4). In addition, the duration of VTs during ischemia and the first 5 min of reperfusion were markedly reduced in the lactating and IR groups as compared with the non-lactating group (p < 0.01, Figure 5).

## Discussion

The current study demonstrates the effect of pregnancy and lactation on myocardial vulnerability to an ischemic insult. We observed that lactation, and not pregnancy alone, preconditioned the maternal heart against ischemia-induced myocardial infarction. Furthermore, nursing reduces the incidence and duration of ventricular arrhythmias during ischemia.

Growing evidence has indicated short-term and long-term beneficial effects of lactation on the risk factors associated with cardiovascular morbidity.^22,23^ The only study indicating cardioprotective effects of lactation against IR injury was recently performed by Shekarforoush and Safari.^17^ However, their study did not take into account if the cardioprotection was conferred by the pregnancy alone. In addition, these authors were unable to observe antiarrhythmic effects of lactation during myocardial ischemia.^17^ In the current study, *in vitro* Langendorff model was used. Thus, the early-onset cardioprotective effects of lactation can be attributed to the intrinsic characteristics of the heart independent of the complex physiology of lactation. We observed that pregnancy increases the ischemia-induced IS and the incidence and duration of arrhythmias. Previous studies suggest that pregnancy enhances the maternal risk of cardiovascular events by increasing central fat accumulation,^24^ blood pressure,^25^ and insulin resistance.^26^ During pregnancy, the maternal heart transforms into "a better functioning heart" and undergoes physiological hypertrophy in order to increase the cardiac pumping capacity. However, Lain et al.^26^ demonstrated that the heart during late pregnancy is more susceptible to IR injury when subjected to coronary occlusion. Furthermore, during late pregnancy in mice, the IS was greater and the post-ischemic functional recovery was found to be extremely poor. Interestingly, the hemodynamic alterations and increased IS were partially restored in the post-partum mice.

The opening of mitochondrial permeability transition pore (mPTP) at the onset of reperfusion is a critical determinant of myocardial cell death.^27^ In this regard, a study demonstrated that pregnancy lowers the threshold for the mPTP opening, which can be attributed to pregnancy-induced increase in cardiac reactive oxygen species (ROS) generation. Some authors have hypothesized that lactation may induce a resetting effect to the heart and improve pregnancy-induced alterations in cardiovascular dynamics.^3^,^28^

The protective effect of lactation may be attributed to an increased metabolic expenditure of a nursing mother,^29^ explaining the decrease in body mass index and cholesterol levels following lactation. In addition, initiation and maintenance of lactation involve many hormones such as oxytocin, prolactin, growth hormone, thyroxine, adrenal corticoids, and parathyroid hormone.^30^ Cardiac tissue expresses a wide variety of hormone receptors, including receptors for lactogenic hormones,^31,32^ and nursing-induced changes in hormonal milieu have been reported to improve the cardiovascular profiles.

Suckling is the major stimulus for the release of oxytocin from the posterior pituitary. The protective effect of oxytocin against IR injury has been previously depicted.^14^ Faghihi et al.^33^ demonstrated that oxytocin preconditioning reduces ischemia-induced ventricular arrhythmias by scavenging free radicals and delaying the opening of mPTP. Das and Sarkar^15^ have suggested an involvement of mitochondrial ATP-sensitive potassium channels in oxytocin -induced cardioprotection. Furthermore, oxytocin has been shown to promote the release of atrial natriuretic peptide - a well-known cardioprotective hormone, which reduces the incidence of reperfusion- induced arrhythmias.^34,35^ Oxytocin also exerts negative inotropic and chronotropic effects^36^ which, in turn may decrease the oxygen demand of the myocardium and produce a smaller infarct following occlusion of the coronary artery. In addition to oxytocin, pretreatment with thyroid hormone has been shown to protect myocardium against lethal ischemia, in a pattern similar to that of ischemic preconditioning.^37^ We observed that lactation reduced the LVDP, RPP, and HR in ischemic animals. This indicates a positive effect of nursing as it reduces myocardial oxygen consumption and improves oxygen supply to demand ratio.

Suckling does not only stimulate the release of oxytocin but also induces the secretion of prolactin (PRL) and adrenocorticotropic hormone, which are essential for galactopoesis.^38^ The experimental evidence for the effect of prolactin on the cardiovascular system is quite limited. A cohort study reported the direct association of prolactin levels with endothelial dysfunction and increased risk of cardiovascular events and mortality.^39^ Conversely, Krzeminski et al.^40^ observed the antiarrhythmic effects of prolactin (isoform) against IR injury. A 15-day prolactin treatment markedly reduced the adrenaline-induced rise in HR, blood pressure, cardiomyocyte necrosis, and granulocyte infiltration in female rats.^41^ Moreover, corticosteriods confer cardioprotection by binding to glucocorticoid receptors.^42^ Glucocorticoids activate the endothelium-derived nitric oxide synthase (eNOS) and exert anti-inflammatory, antiatherogenic, and anti-ischemic effects.^43^

The autonomic system is an important regulator of lactation and milk ejection. Vagal nerve stimulation (VNS) is essential for suckling-induced oxytocin and prolcatin release.^44^ Efferent signals from the vagus nerve can inhibit the production of proinflammtory cytokines, thereby improving the pathological outcomes of diseases like sepsis, myocardial ischemia and other inflammatory disorders.^45^ Interestingly, VNS has been shown to prevent reperfusion injury through inhibition of mPTP.^46^

In the current study, the protective effect of lactogenesis against IR injury can involve the potential role of lactogenic hormones, which can directly influence the cardiac dynamics via cardiac receptors. Furthermore, lactation may have improved the IR injury via VNS. A plausible confounder in this study was the emotional stress imposed on the maternal health due to the separation of the mothers from their pups. Although according to the research protocol the rats in the non-lactated group did not undergo surgery until 21 days after parturition (similar to the rats in the lactating group), the potential effect of emotional stress on cardiovascular dynamics cannot be definitely ruled out.

## Conclusion

Taken together, our findings demonstrate the cardioprotective effects of lactation on maternal health, independent of pregnancy. Moreover, rats which were not allowed to breast-feed their pups, demonstrated high vulnerability to myocardial ischemic insult.
